# Sex-specific aspects of endogenous retroviral insertion and deletion

**DOI:** 10.1186/1471-2148-13-243

**Published:** 2013-11-07

**Authors:** Patrick Gemmell, Jotun Hein, Aris Katzourakis

**Affiliations:** 1 Department of Zoology, University of Oxford, The Tinbergen Building, South Parks Rd, Oxford OX1 3PS, UK; 2 Department of Statistics, University of Oxford, 1 South Parks Road, Oxford,OX1 3TG, UK

## Abstract

**Background:**

We wish to understand how sex and recombination affect endogenous retroviral insertion and deletion. While theory suggests that the risk of ectopic recombination will limit the accumulation of repetitive DNA in areas of high meiotic recombination, the experimental evidence so far has been inconsistent. Under the assumption of neutrality, we examine the genomes of eighteen species of animal in order to compute the ratio of solo-LTRs that derive from insertions occurring down the male germ line as opposed to the female one (male bias). We also extend the simple idea of comparing autosome to allosome in order to predict the ratio of full-length proviruses we would expect to see under conditions of recombination linked deletion or otherwise.

**Results:**

Using our model, we predict the ratio of allosomal to autosomal full-length proviruses to lie between

$\frac{3}{2}$ and $\frac{2}{3}$ under increasing male bias in mammals and between 1 and 2 under increasing male bias in birds. In contrast to our expectations, we find that a pattern of male bias is not universal across species and that there is a frequent overabundance of full-length proviruses on the allosome beyond the ratios predicted by our model.

**Conclusions:**

We use our data as a whole to argue that full-length proviruses should be treated as deleterious mutations or as effectively neutral mutations whose persistence in a full-length state is linked to the rate of meiotic recombination and whose origin is not universally male biased. These conclusions suggest that retroviral insertions on the allosome may be more prolific and that it might be possible to identify mechanisms of replication that are enhanced in the female sex.

## Background

As an obligate part of their life-cycle, retroviruses integrate genetic information into their host’s cellular DNA. If such an integration occurs in a germ line cell and is not sufficiently harmful to its host then it is possible for viral DNA to pass vertically from parent to progeny. Over time, endogenized viral DNA may become fixed within populations and it is therefore possible to detect the traces of ancient viral infections, often as fragments, by trawling modern genomes e.g. [1,2].

Most endogenous retroviruses (ERVs) are not observed in their original full-length proviral form. Immediately after successful integration, a provirus will consist of a pair of long terminal repeats (LTRs) that flank the open reading frames for several retroviral genes, typically *gag*, *pol* and *env*. As flanking LTRs are identical at the time of insertion [3], a persistent similarity between viral extremities over generations means that there is a strong possibility of illegitimate recombination between LTRs from the same or similar ERVs. Recombinational deletion is said to occur when the internal region of a provirus is eliminated by recombination between LTRs and only a solitary (or solo-) LTR is left behind [4]. As ERVs may replicate within the genome via reinfection or retrotransposition [5-7], recombinational deletion is one of the forces shaping both the retention and proliferation of selfish genetic elements [8] and is therefore of interest to those concerned with the accumulation of repetitive DNA over time.

It has previously been shown that recombinational deletion in humans is correlated with local meiotic recombination rate but that the fixation of ERVs is not [9]. These findings are consistent with work examining transposons in worms [10] and retrotransposon specific evidence from flies [11], but are also in contrast to theory and experimental evidence that suggests that transposable elements in general are more frequent in chromosomal regions with lower rates of recombination (see e.g. [12,13]).

In the aforementioned work [9], the authors proposed that the majority of retroviral insertions are acquired down the male germ line due to the relatively high number of cell divisions involved in the production of sperm in the male as opposed to eggs in the female. As many exogenous viruses require cell division in order to cross the nuclear membrane [14], or are at least more efficient at infecting dividing cells [15], it is reasonable to hypothesize that a deeper germ line will offer more opportunities for retroviral infection than a shallow one. This male bias hypothesis was supported by data showing an excess of ERVs on the Y chromosome, even after the chromosome’s low gene density was taken into account [9]. The reasoning behind such a hypothesis is similar to original arguments for male mutational bias [16] in which cell division is associated with error prone DNA replication: in both cases cell division is thought to be correlated with changes in germ line DNA. Although estimates of male mutation bias vary considerably [17], it is generally thought that male bias correlates to life-history traits, with longer lived animals tending to exhibit a higher male bias than shorter lived ones [18].

Previous work on human ERVs [9] is robust but limited in two ways. First, recent evidence suggests that the rate of recombination along the length of chromosomes can vary rapidly [19] and therefore we are not sure how closely recent recombination rates correlate with those in the distant past. Second, we are interested in species beyond humans, including those for which we do not have a recombination map or even an assembly of the Y chromosome. To address these challenges we develop a straightforward model relating recombinational deletion, sex specific ERV integration rates and meiotic recombination at a chromosome level and then use it to examine whether genomic data from several species supports the conclusions of previous work.

### Model

We want to consider how a sex specific ERV integration rate interacts with a recombinational deletion process that is either independent of or strongly linked to the background rate of meiotic recombination. To do so we will consider retroviral insertions under the XY and ZW sex-determination systems. We do this with the intention of comparing the density of ERVs on the allosome (X and Z chromosomes only) to those on the autosome using publically available mammalian and bird genomes.

Assume that retroviral integrations into host genes are highly deleterious or lethal and that the insertions we see today are effectively neutral and fixed by drift. We will consider *p*_*i*_, the proportion of full-length proviruses per unit length of chromosome *i*. We write

$$p_{i}=n_{i}/(l_{i}-g_{i})$$

where *n*_*i*_ is the number of full-length proviruses on chromosome *i*, *l*_*i*_ is the length in base pairs (bp) of chromosome *i*, and *g*_*i*_ is the number of bases on chromosome *i* that are part of a gene. We will use subscript *a* to refer to autosomal DNA and subscript *x* or *z* to refer to allosomal DNA.

Let *f* be the rate of proviral integration for females and let *m*=*βf* be the rate of proviral integration for males, where *β* is a positive real number used to model male bias i.e. the ratio of viral integrations occurring down the male germ line to those viral integrations occurring down the female germ line. As the X chromosome spends twice the amount of time in the female as the male, the average rate of proviral integrations on the X chromosome will be

$$\frac{1}{3}\mathrm{\beta f}+\frac{2}{3}\;f$$

while the average rate of proviral integrations on an autosome will be

$$\frac{1}{2}\mathrm{\beta f}+\frac{1}{2}\;\mathrm{f.}$$

We are interested in knowing whether recombinational deletion is linked to meiotic recombination and whether male bias has been an important factor in integrations. Let *r*_*x*_ and *r*_*a*_ represent the intensity of the process that deletes full-length proviruses from the X chromosome and the autosomes respectively. The rate of accumulation of full-length proviruses on the X chromosome is given by

(1) $${\dot{n}}_{x}=\frac{1}{3}\mathrm{\beta f}+\frac{2}{3}\;f-r_{x}n_{x}$$

while the rate of accumulation of full-length proviruses on an autosome will be

(2) $${\dot{n}}_{a}=\frac{1}{2}\mathrm{\beta f}+\frac{1}{2}\;f-r_{a}n_{a}.$$

We will use the ratio $p=\frac{p_{x}}{p_{a}}$ to make predictions under various scenarios. Equations 1 and 2 have a straightforward analytical solution (Additional file 1) so that in general we have

$$\underset{t\to \infty }{\text{lim}}\frac{n_{x}}{n_{a}}=\frac{r_{a}}{r_{x}}\frac{2}{3}\frac{\left(\beta +2\right)}{\left(\beta +1\right)}.$$

As the X chromosome (excluding pseudoautosomal regions) recombines at only $\frac{2}{3}$ the rate of the autosomes we have $r_{x}=\frac{2}{3}r_{a}$ in the case that the deletion process is linked to meiotic recombination and *r*_*x*_=*r*_*a*_ otherwise. Therefore we arrive at Equation 3 and Equation 4 which give asymptotic values of *p* as a function of *β* in the presence or absence of recombination linked deletion respectively.

(3) $$p=\frac{\left(\beta +2\right)}{\left(\beta +1\right)}$$

(4) $$p=\frac{2}{3}\frac{\left(\beta +2\right)}{\left(\beta +1\right)}$$

**Figure 1:**
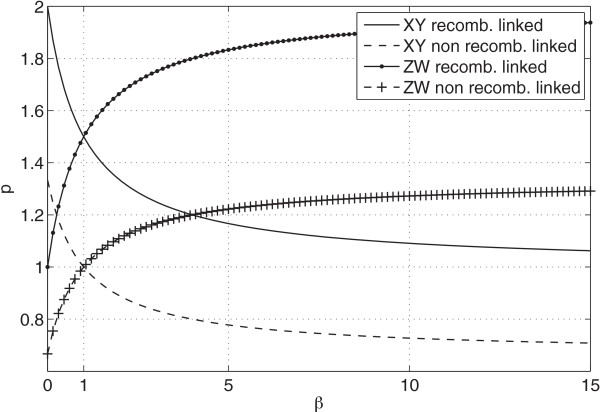
**Predicted ratios.** Predicted ratios of full-length proviruses on the allosome (X or Z) to the autosome under recombination linked and non recombination linked deletion scenarios. Predictions are shown for both the XY sex-determination system and the ZW sex-determination system. For any given bias (*β*) and sex-determination system we make two predictions as to the allosome-to-autosome ratio of full-length proviruses (*p*). A value of *β* greater than one is a male bias and a value of *β* less than one is a female bias. Under the ZW system (e.g. in birds) both male bias and a lack of recombination may contribute to an excess of ERVs on the Z chromosome when compared to the autosome.

These functions are similar to those originated by [16] in the context of molecular evolution and are plotted in Figure 1. In the case that recombination is linked to deletion we expect to see more ERVs on the X chromosome due to its reduced rate of meiotic recombination. As the X chromosome also spends less time in males, the expected excess will be reduced in line with male bias so that as *β* increases the value of *p* will tend to unity. In the case that recombination is not linked to deletion then ERVs on the X chromosome are no more or less effectively deleted by the host. Now we do not expect any excess of ERVs unless male bias is strong, in which case the autosomes will receive more insertions than the X chromosome and *p* will tend to $\frac{2}{3}$ as *β* increases.

In birds, males are the homogametic sex, each having two Z chromosomes, and therefore our model makes different predictions. Under the ZW sex-determination system

$${\dot{n}}_{z}=\frac{2}{3}\mathrm{\beta f}+\frac{1}{3}\;f-r_{z}n_{z}$$

so that by using a similar argument as before *p*_*z*_/*p*_*a*_ is given by

(5) $$p=\frac{\left(2\beta +1\right)}{\left(\beta +1\right)}$$

in the case of recombination linked deletion and

(6) $$p=\frac{2}{3}\frac{\left(2\beta +1\right)}{\left(\beta +1\right)}$$

otherwise. When calculating *p*=*p*_*z*_/*p*_*a*_ male bias and reduced meiotic recombination both act in the same direction to increase the expected excess of ERVs on chromosome Z. As shown in Figure 1, in this case we expect *p* to be $\frac{3}{2}$ tending to 2 as *β* increases when recombination is linked to deletion and *p* to be 1 tending to $\frac{4}{3}$ otherwise.

## Methods

In order to compare our model with reality we obtained an estimate of *p*_*i*_ for the chromosomes of eighteen species. This was done by counting full-length proviruses on each chromosome of the genome of each species and then using gene annotation information to calculate *l*_*i*_ and *g*_*i*_.

Eighteen soft-masked animal genomes were obtained from the Ensembl project [20]: cat, chicken, chimp, cow, dog, gorilla, horse, human, macaque, marmoset, mouse, opossum, orangutan, pig, rabbit, rat, turkey and zebra finch. A collection of 771 viral *pol* sequences was used to locate as many potential endogenized *pol* sequences as possible from across all eighteen genomes. The 771 probe sequences were selected to represent the full diversity of exogenous and endogenous retroviral genes and are the same as those used in previous studies [1,21]. Application of tBLASTn [22] identified putative *pol* hits which were used to extract 49,928 non-overlapping 15kb regions each centred on a match. These 49,928 regions were processed using LTRharvest [23], a tool designed to detect full-length LTR retrotransposons based on structural features alone. Thus, a large set of tBLASTn results were reduced to 18,203 putative full-length sequences of which we filtered the 16,661 that occur in sequence that is assembled into chromosomes of interest.

For some genomes LTRharvest was inclined to report sequences made up of a large amount of unknown nucleotide sequence, sequence recorded with Ns in the genome, as retrotransposon like. These Ns between LTRs are doubly problematic as they lead us to question whether LTRs are genuinely physically associated and also make it harder to confirm that the internal regions contain viral genes. To be more certain that we were dealing with genuine full-length proviruses we discarded any sequence containing more than five consecutive unknown nucleotides or comprising more than five percent unknown nucleotides overall. These particular cutoff values are conservative and were chosen with caution in mind. We then used the LTRdigest annotation tool [24] to further discard any full-length proviral sequences that did not contain at least one retro-viral gene beyond the *pol* previously identified by homology. This filtering process left 7,299 full-length sequences for analysis as is recorded in Additional file 2.

From Ensembl genes69 we estimated *g*_*i*_ for each chromosome of interest using the BioMart section of the website. As gene annotations can overlap we post-processed the downloaded results to ensure that each base pair of annotation contributed at most once by using an algorithm that incrementally merged overlapping annotations. The total length of each chromosome *l*_*i*_ was available both from Ensembl and also directly from the genomes themselves. Each putative provirus occurring on known chromosomal DNA contributed to the total count *n*_*i*_ for the chromosome.

As we are interested in any overall bias in retroviral insertions we also performed a survey of solo-LTRs across all eighteen genomes. In this case we compiled a query library containing the 5’ LTR region of each of the 7,299 full-length proviruses and performed a BLASTn search against every genome. Alignments of at least 95% identity and covering at least 95% of the query sequence were retained and multiple overlapping alignments were merged to give 926,894 putative LTRs. As the purpose of this search was to identify solo-LTRs, any putative LTRs separated by less than 15kb of intermediate sequence were discarded leaving 508,811 merged alignments that we consider represent the solitary remnants of proviruses.

We use solo-LTRs as a proxy for total insertions which is justifiable given the fact that they are so much more numerous than full-length proviruses, as is recorded in Additional file 3. However, as we detect solo-LTRs based solely on their similarity to LTRs of full-length proviruses we will not identify solo-LTRs that have no full-length proviral representatives.

For both full-length proviruses and solo-LTRs, we also checked that the ratio of allosomal (X or Z) to autosomal ERVs is not correlated to GC content (Additional file 4).

## Results and discussion

**Figure 2:**
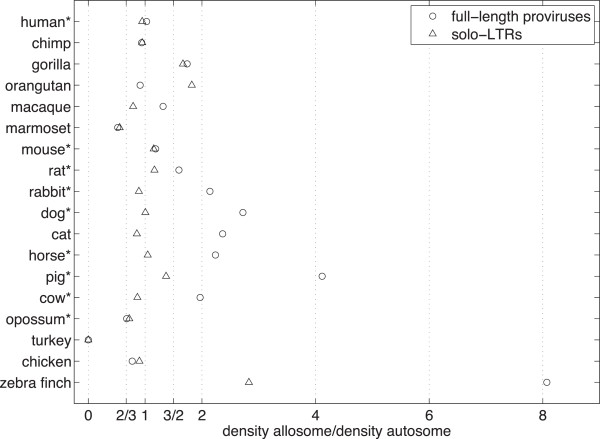
**Observed ratios.** Observed ratios of full-length proviruses and solo-LTRs on the allosome (X or Z) to the autosome for the genomes of 15 mammals and 3 birds. Vertical lines mark the key ratios $\frac{2}{3}$, 1, $\frac{3}{2}$ and 2. Asterisks mark the genomes we consider as trustworthy and discuss in the Results section. Mammalian full-length provirus ratios typically lie beyond 3/2, the maximum predicted value under an assumption of male-bias. Mammalian solo-LTRs are generally more evenly distributed between autosome and allosome. Mammalian solo-LTRs are also generally relatively less abundant on the allosome than full-length proviruses.

We wish to see whether the predictions of our model are borne out by genomic data and so for eighteen genomes we aggregate retroviral insertions into two groups for easy display and analysis: those on the allosome (X or Z) and those on the autosome. That is to say, the ratio *p* is estimated by calculating $n_{a}=\sum_{i}n_{i}\;\text{such that}\;i\notin \{x,y,w,z\}$ and using *n*_*x*_ or *n*_*z*_ as appropriate. We plot the ratio *p* in Figure 2 and present the raw data in Additional file 3.

Under our model, every ratio *p* implies two bias values *β*, one for each deletion scenario. For each genome studied we make three point estimates of *β*, one based on solo-LTR ratios and two based on full-length proviral ratios under the scenario of recombination linked deletion and otherwise. We record the results in Table 1.

**Table 1:** Point estimates and intervals on bias

					**Mammals**				
	**solo-LTRs**			**Full-length recomb.**			**Full-length no recomb.**		
	**point**	**lower**	**upper**	**point**	**lower**	**upper**	**point**	**lower**	**upper**
human*	1.36	1.03	1.79	38.24	2.56	*∞*	0.86	0.09	3.32
chimp	1.36	0.89	2.08	NA	1.48	*∞*	1.44	0	*∞*
gorilla	NA	0	0.05	0.35	0	16.13	NA	0	0.7
orangutan	NA	NA	NA	NA	0.63	*∞*	1.73	0	*∞*
macaque	4.47	3.35	6.28	2.18	0.13	*∞*	0.03	0	1.65
marmoset	NA	NA	NA	NA	3.39	*∞*	NA	0.19	*∞*
mouse*	0.37	0.28	0.48	4.39	1.73	33.87	0.29	0	0.84
rat*	0.34	0.23	0.46	0.68	0	3.74	NA	0	0.23
rabbit*	1.99	1.53	2.62	NA	0	2.85	NA	0	0.12
dog*	0.97	0.78	1.2	NA	0	1.79	NA	NA	NA
cat	2.56	1.17	7.12	NA	0	2.02	NA	0	0
horse*	0.76	0.01	3.18	NA	0	4.98	NA	0	0.33
pig*	NA	0	0.07	NA	NA	NA	NA	NA	NA
cow*	2.38	2.02	2.82	0.03	0	1.06	NA	NA	NA
opossum*	11.22	5.12	167.79	NA	4.39	*∞*	77.19	0.28	*∞*
					**Birds**				
	**point**	**lower**	**upper**	**point**	**lower**	**upper**	**point**	**lower**	**upper**
turkey	NA	0	*∞*	NA	0	*∞*	NA	0	*∞*
chicken	0.53	0	*∞*	NA	0	*∞*	0.19	0	*∞*
zebra finch	NA	NA	NA	NA	NA	NA	NA	NA	NA

Point estimates and intervals on bias *β* implied by measurement of: solo-LTR distribution (left); full-length proviral distribution under the recombination linked deletion model (middle); full-length proviral distribution under the non recombination linked deletion model (right). Although each model implies a single bias *β*, we also ask what bias values delineate the range (lower and upper) under which we could expect to measure our observed ratios with a probability of less than 0.05. We use ‘NA’ to mark those situations in which no point estimate or boundary value of *β* can be computed. Asterisks mark the genomes we consider as trustworthy and discuss in the Results section.

As our point estimates would vary if we had counted differing numbers of viruses on the autosome or allosome, we also use equations from the Model section to identify the range of bias outside of which our ratios would be observed with probability less than 0.05. We do this by solving our equations for *n*_*x*_ or *n*_*z*_, the number of viruses expected on the allosome, and then finding the range of *β* for which the Chi-square statistic is less than 3.841, the 0.05 p-value for a 1 d.o.f. goodness of fit (see Additional files 5 and 6). Where *β* would be less than 0 we consider a prediction non-applicable (NA).

Three aspects of our results are immediately striking. First, the ratio of full-length proviruses and solo-LTRs shows a great deal of variation between species, with both over and under abundance on the allosome represented in our results. Second, solo-LTR ratios tend to fall within the range $\frac{2}{3}$ to $\frac{4}{3}$ that make sense in the context of our model. Third, our results show that full-length proviruses are more abundant on the allosome than the autosome with the exception of orangutan, opossum, marmoset, chimp and chicken. We elaborate on these three observations in turn below.

### LTR detection, genome variation and phylogenetic independence

Our ERV counting methodology relies on de-novo LTR retrotransposon detection. We favour the approach in this study as we expect de-novo prediction to work well on both familiar and lesser studied ERVs, an important consideration when we examine genomes that are relatively distant from humans. It is important to note that we do not expect to detect all full-length proviruses but merely to detect a consistent proportion across either allosomal or autosomal DNA. A similar expectation holds for false positives: our requirement of our tool is consistency.

The actual number of proviruses we use in our study is less than can be detected in principle and our results can be improved by using more effective counting methods. In the present study we are keen to retain a structural model for ERVs and therefore reject the use of repeat masking tools that are designed to detect repetitive nucleotide sequences, that may well be fragmented, rather than accurately count individual proviruses. We proceed with the knowledge that studies combining the framework from the Model subsection with better tools may well provide better estimates of bias. We certainly do not consider the data we use exhaustive but do think it a reasonable sample.

The genomes we examine vary in the number of ERVs they contain but also in how often we are willing to trust the results we obtain from them. For example, LTRharvest identifies 1,228 ERVs in the orangutan genome but we must throw roughly two-thirds away because they contain many consecutive unknown nucleotides (Ns); for the dog we identify just 177 ERVs but need discard only six percent. For this reason we consider the genomes of the cow, dog, horse, human, mouse, opossum, pig, rabbit and rat as trustworthy for our purposes as we discard less than one third of potential proviral sequences due to unknown nucleotides. We consider the remaining nine genomes less informative as the opposite is the case and we are particularly disappointed that so few full-length proviruses could be recovered from bird genomes. We largely restrict the remainder of our discussion to results from the more trustworthy genomes and mark those genomes with asterisks when appearing in tables or figures.

We do not perform a phylogenetic analysis on our results as we know that most full-length proviruses are not shared among species as closely related as human and macaque (for example, 70% of full-length sites in macaque are not present in solo or full-length form in any of human, chimp, gorilla or orangutan based on our own unpublished analysis) and because we do not draw conclusions that involve making detailed comparison between species. Rather, we examine a diverse set of animal genomes and recognise that some applications of our method, such as those on the primates, are partially pseudoreplicates that produce non-independent results.

The heterogeneity of ERV replication affinity in a genome may be a confounding factor in our study. If some types of virus are better at infecting male germ line cells and others are better at infecting female germ line cells then the former variety will show more male bias than the latter variety. As various kinds of ERVs may have different biases it is important to note that our model treats bias (and rates of proviral insertion and deletion) as averages. Similarly, as the ERVs in a genome are only derived from a relatively small number of ERV lineages, any replication affinity of particular lineages could, in principle, bias the result. For example, in humans one-third of all ERVs are descended from thirty-one to forty distinct colonizations [3,25].

### Ratios of Solo-LTRs

Using the results from our more informative genomes we want to address the role of male bias and recombination linked deletion in ERV biology. Our intervals for solo-LTR biases are tight (Table 1) and our results suggest that cow, human, opossum and rabbit all have a male biased insertion history. On the other hand, mouse and rat exhibit a female biased insertion history while dog and horse give ambiguous results. These results are surprising because, as discussed in the introduction, we expect ERV integration bias to be male oriented and positively correlated with generation time in the same way that mutational bias is.

Here our results appear to unlink deep germ lines and ERV proliferation in general, perhaps suggesting that ERV integration tends to take place during a short window of time that is unrelated to the protracted process of germ line cell division. We also think it possible that integrations might be driven by ERV expression in placental tissue [26,27]. While transmission from or via placenta to progeny will affect both sexes of embryo equally, placental tissue could also re-infect maternal germ line cells. Therefore placental expression of ERVs could well have the effect of reducing male bias overall. The effect would be stronger for species in which the females spend a greater proportion of their lives bearing offspring.

Nevertheless, it is generally thought that conventional mutational male bias should increase with generation time, metabolic rate and sperm competition. A comprehensive study [18] used age at sexual maturity, maximum life span, and interlitter interval as proxies for generation time; basal metabolic rate, body mass and body temperature as proxies for metabolic rate; and testes-to-body mass ratio and mating patterns (polyandrous/polygynandrous versus monogamous/polygynous) as proxies for sperm competition. The conclusion was that that generation time was a powerful predictor of mutational bias but that metabolic rate was of less use; sperm-competition appeared to be unexplanatory [18]. While we would not necessarily expect the same results it could be argued that all of the above factors should also be positively correlated with ERV bias. The availability of closely related animal genomes means there is potential for an analogous study of the effects of life-history traits on ERVs.

### Ratios of full-length proviruses

Our results for full-length proviruses are interesting in the extent to which ERVs are over represented on the allosomes. We expect to see ratios in the range $\frac{3}{2}$ to 1 or 1 to $\frac{2}{3}$, which correspond to scenarios of male bias under recombinational deletion or otherwise. Instead, what we observe is that, among our more informative genomes, all ratios besides those for the human and opossum lie beyond the range of values predicted by our model. (We note that the opossum X chromosome is unusual in that it receives *more* recombination than the autosomes [28].) This does not mean our model is useless but instead that we must examine it more closely in order to interpret our results. Therefore we consider three general reasons that we might see an overabundance of proviruses on the allosome: dynamics, a lack of neutrality or stochasticity.

First, ratios close to the asymptotes of our model may not have been reached. Under a recombination-linked deletion scenario it is mathematically possible for LTRs to accumulate on the allosome while recombination ‘catches up’ and restores our predicted ratios. Although we would eventually expect to see the ratios described in the Model section enough time may not yet have passed that we actually do so. This explanation highlights a limitation of our model that can not be addressed solely by examining older proviral insertions.

Second, we may be mistaken in assuming that full-length proviruses are effectively neutral and drift to fixation. In this case factors including linkage disequilibrium, differing mutation rates between sexes, the reduced relative effective population size of the allosome, the heterozygosity of proviral mutations or sexual antagonism mean that we cannot say whether we would expect to see higher or lower ratios than our neutral model predicts.

For example, considering mammals, we expect the female nucleotide substitution rate to be lower than the male substitution rate, in which case proviruses on the X chromosome will receive fewer nucleotide substitutions than those on the autosome. Therefore, we expect that proviruses on the autosome are more likely to be made benign by random mutation than those on the X chromosome and thus are more likely to reach high frequency or fixation via drift or draft. Furthermore, as proviruses will initially be found at low frequencies, any harmful recessive effects will be felt most strongly in the hemizygous sex [29] and so selection against proviruses may be more effective on the X chromosome, also enhancing the relative number of proviruses we might expect to see on the autosome. Both these effects would act in the same direction and increase apparent male bias.

However, the extent to which the above effects hold is not known. For example, while ectopic recombination might be a major harmful consequence of carrying a proviral insertion, it is an open question as to whether it is generally healthier for a host to be homozygous for a proviral insertion or whether other factors dominate; is an ERV best modelled as a recessive harmful mutation? Furthermore, any sexual antagonism in the effects of proviral insertions can shift our expectations of relative abundance of proviruses in either direction. For example, we would expect to see more fixation of proviruses on X for recessive mutations that are of benefit to males but harmful to females or for dominant mutations that are of benefit to females but harmful to males [30]. Dominance and antagonism effects are examples of unknown factors that can decrease any apparent male bias or lead to an apparent female bias instead.

Overall, these complexities are such that we cannot incorporate selection into our framework without knowing more about the harm full-length proviruses cause. The explanation that ERVs are non-neutral implies a misapplication of our model and might possibly be supported by the recent observation that in mouse there are about 75% of the expected number of polymorphic (unfixed) ERVs on the X chromosome yet close to the expected amount of fixed ones [31]. As an apparent underabundance of TEs on the X chromosome is reduced over time this evidence suggests that polymorphic ERVs are more likely to fix on the X chromosome than the autosome. In this case we note that if solo-LTRs are also deleterious, or if the process of proviral deletion often occurs when proviruses are fixed or at a high frequency, then our estimates of bias obtained from solo-LTRs will also be an underestimate.

Third, our results may genuinely reflect the processes described by our model in many cases. On a species-by-species basis the overrepresentation of full-length proviruses that we see on the X chromosome is often statistically compatible with a range of positive bias under both recombination linked and non recombination linked deletion. As Table 1 shows, under the recombination linked scenario fourteen of the fifteen observed ratios are statistically acceptable and ten of the fifteen have a bias that is compatible with that obtained from the corresponding solo-LTR ratios. Under the non recombination linked scenario twelve of the fifteen ratios are statistically allowable and eight of these are compatible with the corresponding solo-LTR ratios. We acknowledge that we find these wide intervals an uncomfortable shortcoming of an approach relying on comparing a small allosome to a large autosome.

Of these three explanations, the first two are applicable in the case that a lack of recombination is in one way or another responsible for the overabundance of full-length proviruses that we highlighted above. With respect to the third explanation, we find our observations are more often suggestive of recombination linked deletion than otherwise. Given we know that opossum X chromosome biology is unusual [28], and also that when our ratios are statistically problematic they tend to be too large, it is reasonable for us to favour a scenario of recombination linked deletion and to question the assumption that ERVs are neutral alleles.

Of course, we may also see ratios that fall outside of our range of predictions as a reason to reject our model entirely, in which case we are obliged to look for some other explanation of what exactly it is about the X chromosome that results in full-length proviruses being more abundant there. Nevertheless, in either case, if full-length proviruses can persist for longer on the X chromosome then it is likely that if we look more closely we will find that viruses that integrate there are more successful replicators than those that arrive elsewhere.

## Conclusions

We predicted the allosomal to autosomal ratio of full-length proviruses we would expect to see under a neutral model given recombination linked deletion or otherwise. Using bioinformatics tools we detected an excess of full-length endogenous retroviruses on the sex chromosomes of eleven mammals and one bird. We also observed overall patterns of endogenous retroviral abundance that, under a neutral model, are not universally male biased. We suggest that a recombination linked deletion process or non-neutral alleles best explain our observations and that, in future, population data and a better quantification of the harm caused by full-length proviruses can help us more accurately explain their relative frequencies on sex chromosomes.

## Competing interests

The authors declare that they have no competing interests.

## Authors’ contributions

PG and AK conceived the project. PG developed the models and performed the analysis with supervision from AK and JH. PG, JH and AK wrote the paper. All authors read and approved the final manuscript.

## Supplementary Material

Additional file 1 **Equations.** Note on asymptotic ratios. Click here for file

Additional file 2 **Filtering.** For autosome and allosome, counts of full-length proviruses that are found and filtered based on unknown nucleotides and annotation by LTRdigest. Click here for file

Additional file 3 **Raw data.** Cross species post-filtering ERV, solo-LTR and bps counts used in analysis. Click here for file

Additional file 4 **GC content.** Plots illustrating that the ratios of full-length proviruses and solo-LTRs are not correlated with the ratio of GC content. Click here for file

Additional file 5 **Point estimates and intervals.** Details of the formulae used to calculate point estimates of bias and the bias intervals outside of which our observed autosomal to allosomal ratios would occur with probability less than 0.05. Click here for file

Additional file 6 **Graphical representation of bias point estimates and intervals.** Plots of point estimates of bias and the bias intervals outside of which our observed autosomal to allosomal ratios would occur with probability less than 0.05. Click here for file
