# Supplementary material for: Sex-specific aspects of endogenous retroviral insertion and deletion
Source: BMC Evol Biol. 2013 Nov 7;13:243. doi: 10.1186/1471-2148-13-243 (PMC3827010; doi:10.1186/1471-2148-13-243)
Supplement: Additional file 1 — Equations. Note on asymptotic ratios. [file 1471-2148-13-243-S1.pdf]

Additional file 1 — equations

In general,

$$\dot{n_i} = a + bn_i \implies n_i = \frac{e^{bt}[a + bn_i(0)] - a}{b}$$

so that

$$\frac{n_i}{n_j} = \frac{b_j}{b_i}\frac{e^{b_i t}[a_i + b_i n_i(0)] - a_i}{e^{b_j t}[a_j + b_j n_j(0)] - a_j}$$

and when $b_i < 0$ and $b_j < 0$ and $a_j \neq 0$

$$\lim_{t\to\infty} \frac{n_i}{n_j} = \frac{b_j}{b_i}\frac{a_i}{a_j}. \tag{1}$$

In the case of the mammals we let $a_i = \frac{1}{3}m + \frac{2}{3}f$ and $a_j = \frac{1}{2}m + \frac{1}{2}f$ in Equation 1 while in the case of birds we let $a_i = \frac{2}{3}m + \frac{1}{3}f$. For recombination linked deletion we substitute $b_i = \frac{2}{3}b_j$ otherwise we substitute $b_i = b_j$.
