# Supplementary material for: Sex-specific aspects of endogenous retroviral insertion and deletion
Source: BMC Evol Biol. 2013 Nov 7;13:243. doi: 10.1186/1471-2148-13-243 (PMC3827010; doi:10.1186/1471-2148-13-243)
Supplement: Additional file 2 — Filtering. For autosome and allosome, counts of full-length proviruses that are found and filtered based on unknown nucleotides and annotation by LTRdigest. [file 1471-2148-13-243-S2.pdf]

Additional file 2: filtering

		autosome		X		autosome	X	overall
		total	filtered	total	filtered	retained	retained	retained
mammals	human	1630	0	139	0	100%	100%	100%
	chimp	1050	383	74	26	64%	65%	64%
	gorilla	922	621	83	46	33%	45%	34%
	orangutan	1135	745	93	68	34%	27%	34%
	macaque	1011	395	90	33	61%	63%	61%
	marmoset	516	224	32	19	57%	41%	56%
	mouse	3264	3	310	1	100%	100%	100%
	rat	1092	297	124	36	73%	71%	73%
	rabbit	399	62	40	8	84%	80%	84%
	dog	160	11	17	0	93%	100%	94%
	cat	263	97	27	11	63%	59%	63%
	horse	150	13	21	0	91%	100%	92%
	pig	198	33	36	4	83%	89%	84%
	cow	820	101	114	24	88%	79%	87%
	opossum	2375	189	31	1	92%	97%	92%

		autosome		Z		autosome	Z	overall
		total	filtered	total	filtered	retained	retained	retained
birds	turkey	52	31	4	4	40%	0%	38%
	chicken	124	64	24	20	48%	17%	43%
	zebra finch	162	104	79	47	36%	41%	37%

Table 1: Full-length proviruses that are found and filtered based on unknown nucleotide sequence (sequence recorded with Ns). For autosome and allosome (X or Z) and for mammals and birds, the table contains the following columns: total (total number of full-length proviruses found); filtered (number of proviruses with more than five consecutive unknown nucleotides or comprising more than five percent unknown nucleotides overall). The table also shows totals retained as percentages.

		autosome		X		autosome	X	overall
		total	annotated	total	annotated	retained	retained	retained
mammals	human	1630	1135	139	83	70%	60%	69%
	chimp	1050	587	74	39	56%	53%	56%
	gorilla	922	477	83	33	52%	40%	51%
	orangutan	1135	530	93	36	47%	39%	46%
	macaque	1011	652	90	55	64%	61%	64%
	marmoset	516	263	32	14	51%	44%	51%
	mouse	3264	2164	310	209	66%	67%	66%
	rat	1092	620	124	75	57%	60%	57%
	rabbit	399	157	40	18	39%	45%	40%
	dog	160	53	17	9	33%	53%	35%
	cat	263	136	27	23	52%	85%	55%
	horse	150	81	21	10	54%	48%	53%
	pig	198	119	36	27	60%	75%	62%
	cow	820	451	114	70	55%	61%	56%
	opossum	2375	795	31	13	33%	42%	34%

		autosome		Z		autosome	Z	overall
		total	annotated	total	annotated	retained	retained	retained
birds	turkey	52	9	4	1	17%	25%	18%
	chicken	124	30	24	4	24%	17%	23%
	zebra finch	162	72	79	45	44%	57%	49%

Table 2: Full-length proviruses that are found and annotated as containing retroviral genes by LTRdigest. For autosome and allosome (X or Z) and for mammals and birds, the table contains the following columns: total (total number of full-length proviruses found); annotated (total number of proviruses with at least one viral gene beyond *pol*). The table also shows totals retained as percentages.
