# Supplementary material for: Sex-specific aspects of endogenous retroviral insertion and deletion
Source: BMC Evol Biol. 2013 Nov 7;13:243. doi: 10.1186/1471-2148-13-243 (PMC3827010; doi:10.1186/1471-2148-13-243)
Supplement: Additional file 3 — Raw data. Cross species post-filtering ERV, solo-LTR and bps counts used in analysis. [file 1471-2148-13-243-S3.pdf]

Additional file 3 — raw data

		autosome					X				
		bps	annotated	ERVs	solo-LTRs	LTRs	bps	annotated	ERVs	solo-LTRs	LTRs
mammals	human	2881033286	1492714527	1135	27372	36133	155270560	56269176	83	1852	2582
	chimp	2963472556	944374562	423	13187	15813	156848144	29895898	25	787	941
	gorilla	2763641886	905908126	151	630	726	154045127	33732766	17	68	80
	orangutan	3204312304	952004569	233	12797	17993	166640359	39307072	12	1318	1905
	macaque	2709717664	918557380	383	52648	97231	153947521	40118004	32	2638	4519
	marmoset	2625311106	985119290	151	42081	63731	142054208	37153777	5	1483	1972
	mouse	2462745373	980225775	2163	49133	105282	171031299	50190099	209	4612	11515
	rat	2558181645	762301001	479	36302	59479	160699376	29104761	56	3098	5726
	rabbit	2136051329	583258565	120	38736	56251	111700775	21064026	15	2012	2882
	dog	2203764842	808527551	51	49812	84301	123869142	33364030	9	3245	5718
	cat	2302113297	675873955	88	4366	4616	126427096	24819152	13	233	245
	horse	2242939370	700386722	71	1108	1151	124114077	26983855	10	73	79
	pig	2450713522	610661496	103	15637	17873	144288218	35762619	25	1262	1542
	cow	2512082506	750482798	402	94393	250256	148823899	26329700	55	5670	15166
	opossum	3423037129	1131125989	709	40820	59373	79335909	21883948	12	738	972

		autosome					Z				
		bps	annotated	ERVs	solo-LTRs	LTRs	bps	annotated	ERVs	solo-LTRs	LTRs
birds	turkey	959032393	300974881	3	3	6	81011772	12746772	0	0	0
	chicken	959567819	383324487	15	142	185	74948554	25293314	1	11	14
	zebra finch	980649417	341792528	24	441	506	75831218	23056320	16	103	130

Table 1: Cross species post-filtering ERV, solo-LTR and bps counts used in analysis. For autosome and allosome (X or Z) and for mammals and birds, the table contains the following columns: bps (total bps of autosome or allosome); annotated (total distinct bps covered by a gene annotation); ERVs (total number of full-length proviruses); solo-LTRs (total number of solo-LTRs at least 15k bps away from another solo-LTR); LTRs (LTR hits, regardless of separation).
