# Supplementary material for: Sex-specific aspects of endogenous retroviral insertion and deletion
Source: BMC Evol Biol. 2013 Nov 7;13:243. doi: 10.1186/1471-2148-13-243 (PMC3827010; doi:10.1186/1471-2148-13-243)
Supplement: Additional file 4 — GC content. Plots illustrating that the ratios of full-length proviruses and solo-LTRs are not correlated with the ratio of GC content. [file 1471-2148-13-243-S4.pdf]

Additional file 4 — GC content

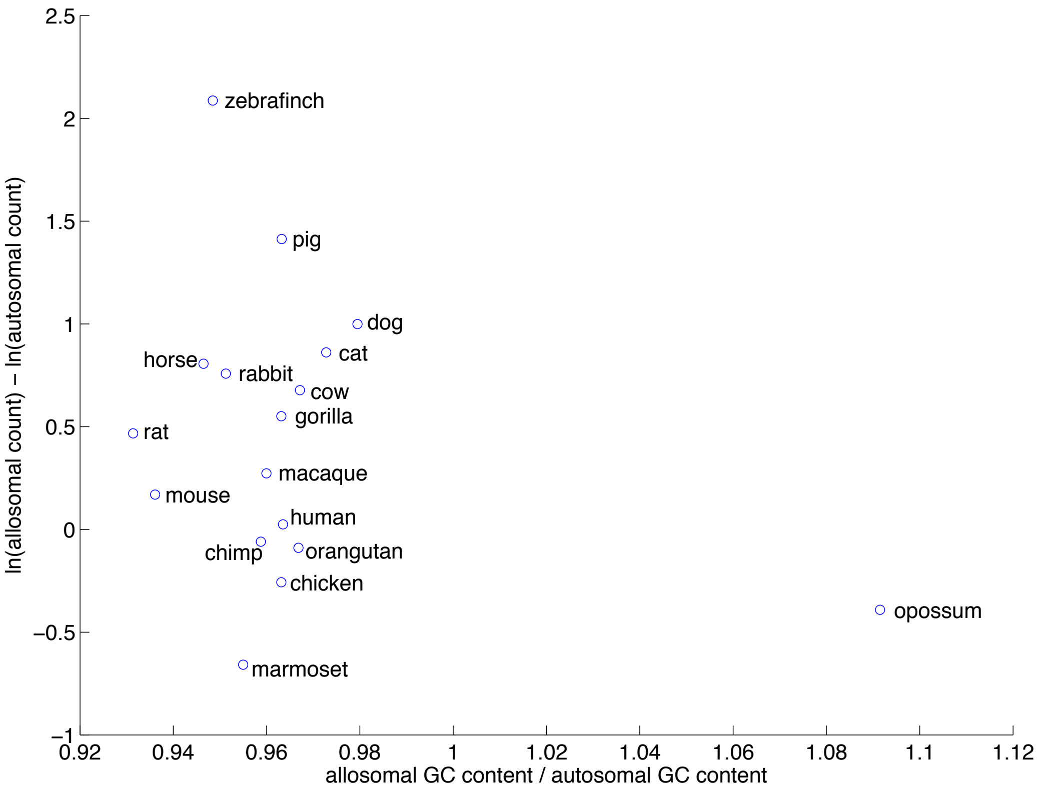

Figure 1: The ratio of allosomal (X or Z) to autosomal full-length proviruses (p) is not correlated with the ratio of allosomal to autosomal GC content.

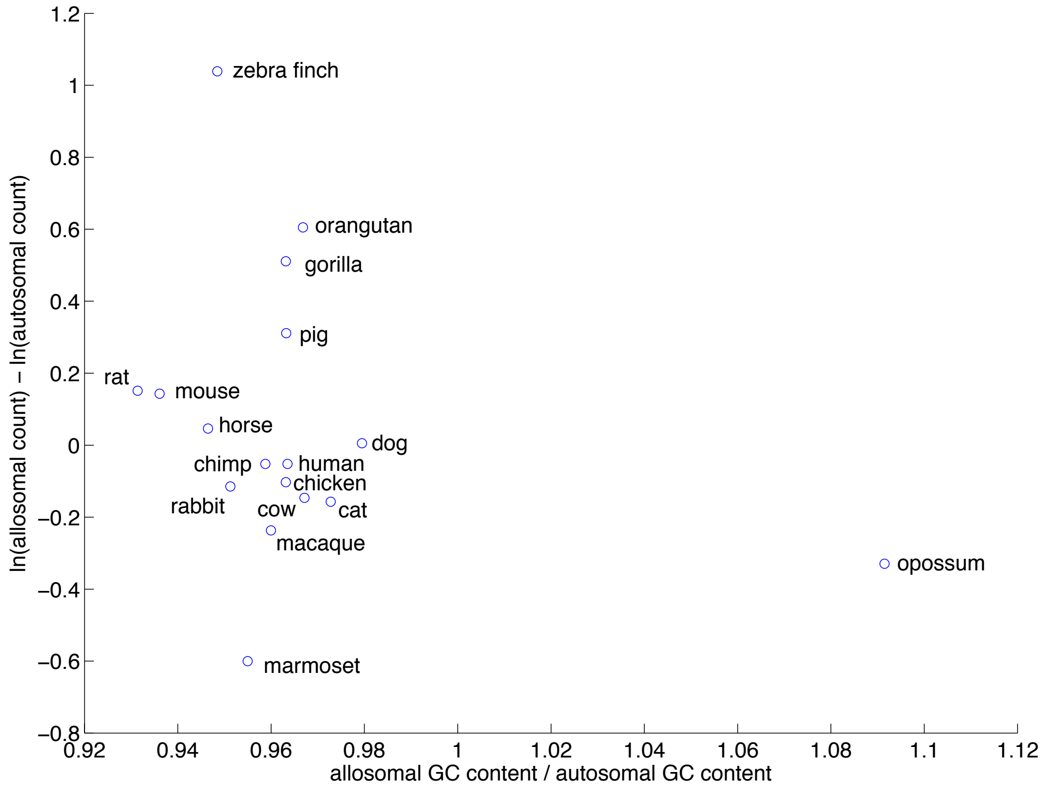

Figure 2: The ratio of allosomal (X or Z) to autosomal solo-LTRs is not correlated with the ratio of allosomal to autosomal GC content.
