# Supplementary material for: Sex-specific aspects of endogenous retroviral insertion and deletion
Source: BMC Evol Biol. 2013 Nov 7;13:243. doi: 10.1186/1471-2148-13-243 (PMC3827010; doi:10.1186/1471-2148-13-243)
Supplement: Additional file 5 — Point estimates and intervals. Details of the formulae used to calculate point estimates of bias and the bias intervals outside of which our observed autosomal to allosomal ratios would occur with probability less than 0.05. [file 1471-2148-13-243-S5.pdf]

Additional file 5 — point estimates and intervals

Mammal solo-LTRs and full-length proviruses under non recombination linked deletion.

Let $b_x = l_x - g_x$, $b_a = l_a - g_a$ and $n = n_x + n_a$ then

$$\text{expected } n_x = \frac{2(2+\beta)b_x n}{3(1+\beta)b_a + 2(2+\beta)b_x}$$

and

$$\beta = \frac{3b_a x + 4b_x(x-n)}{2b_x(n-x) - 3b_a x}.$$

Mammal full-length proviruses under recombination linked deletion.

Let $b_x = l_x - g_x$, $b_a = l_a - g_a$ and $n = n_x + n_a$ then

$$\text{expected } n_x = \frac{(2+\beta)b_x n}{(1+\beta)b_a + (2+\beta)b_x}$$

and

$$\beta = \frac{b_a x + 2b_x(x-n)}{b_x n - (b_a + b_x)x}.$$

Bird solo-LTRs and full-length proviruses under non recombination linked deletion.

Let $b_z = l_z - g_z$, $b_a = l_a - g_a$ and $n = n_z + n_a$ then

$$\text{expected } n_z = \frac{2(1+2\beta)b_z n}{3(1+\beta)b_a + 2b_z + 4\beta b_z}$$

and

$$\beta = \frac{3b_a z + 2b_z(z-n)}{4b_z(n-z) - 3b_a z}.$$

Bird full-length proviruses under recombination linked deletion.

Let $b_z = l_z - g_z$, $b_a = l_a - g_a$ and $n = n_z + n_a$ then

$$\text{expected } n_z = \frac{(1+2\beta)b_z n}{b_a + \beta b_a + b_z + 2\beta b_z}$$

and

$$\beta = \frac{(b_a + b_z)z - b_z n}{2b_z n - (b_a + 2b_z)z}.$$

Calculating intervals for β.

As expected $n_a = n -$ expected n_x (or expected n_z as appropriate) we can calculate a χ^2 value for our observations as a function of β. By finding the positive roots of $\chi^2(\beta) - 3.841$ (where 3.841 is the critical χ^2 value associated with a p-value of 0.05 under a 1 d.o.f. goodness of fit) we can identify the interval of β outside of which our observed ratios would be unlikely to occur due to chance.
