# Supplementary material for: Sex-specific aspects of endogenous retroviral insertion and deletion
Source: BMC Evol Biol. 2013 Nov 7;13:243. doi: 10.1186/1471-2148-13-243 (PMC3827010; doi:10.1186/1471-2148-13-243)
Supplement: Additional file 6 — Graphical representation of bias point estimates and intervals. Plots of point estimates of bias and the bias intervals outside of which our observed autosomal to allosomal ratios would occur with probability less than 0.05. [file 1471-2148-13-243-S6.pdf]

Additional file 6 — graphical representation of bias point estimates and intervals

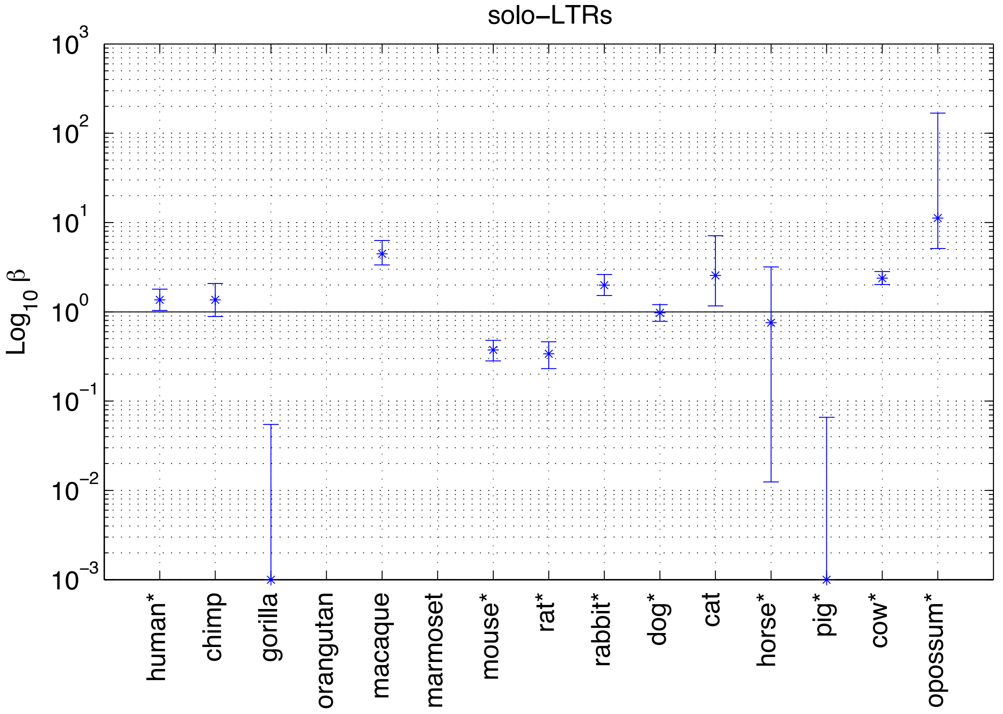

Figure 1: Point estimates and intervals on bias β implied by measurement of solo-LTRs distribution. Points on the graph indicate the single bias β implied by our model. Error bars delineate the range of bias values under which we could expect to measure our observed ratios with a probability of less than 0.05. In those situations in which no point estimate or range of bias can be computed no markings appear. Asterisks beside species mark the genomes we consider as trustworthy and discuss in the Results section.

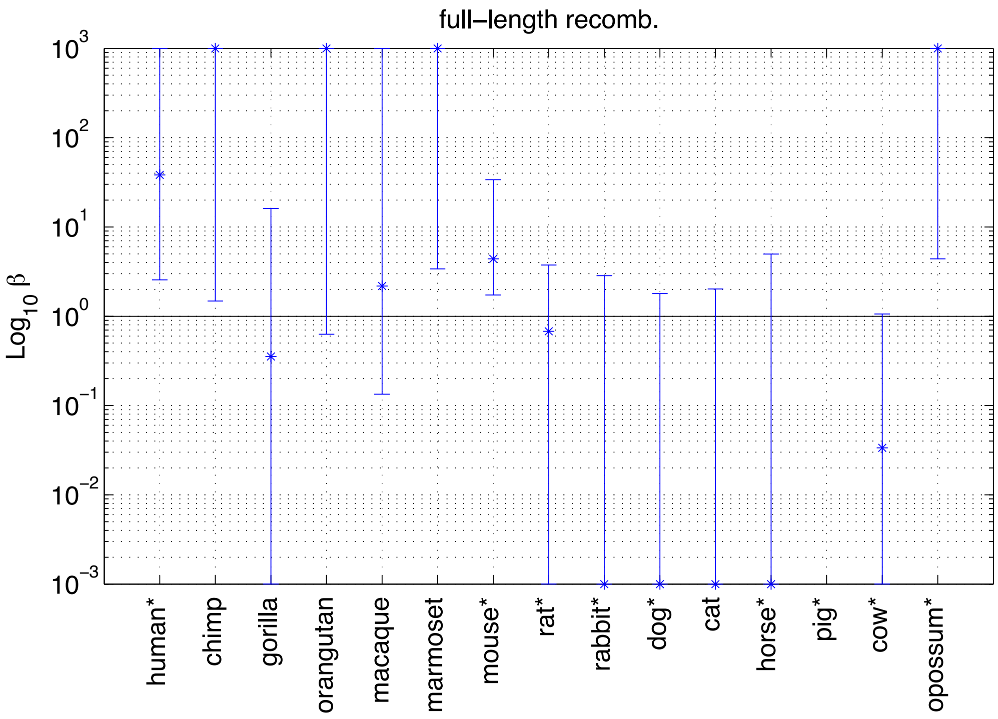

Figure 2: Point estimates and intervals on bias β implied by measurement of proviral distribution under the recombination linked scenario. Points on the graph indicate the single bias β implied by our model. Error bars delineate the range of bias values under which we could expect to measure our observed ratios with a probability of less than 0.05. In those situations in which no point estimate or range of bias can be computed no markings appear. Asterisks beside species mark the genomes we consider as trustworthy and discuss in the Results section.

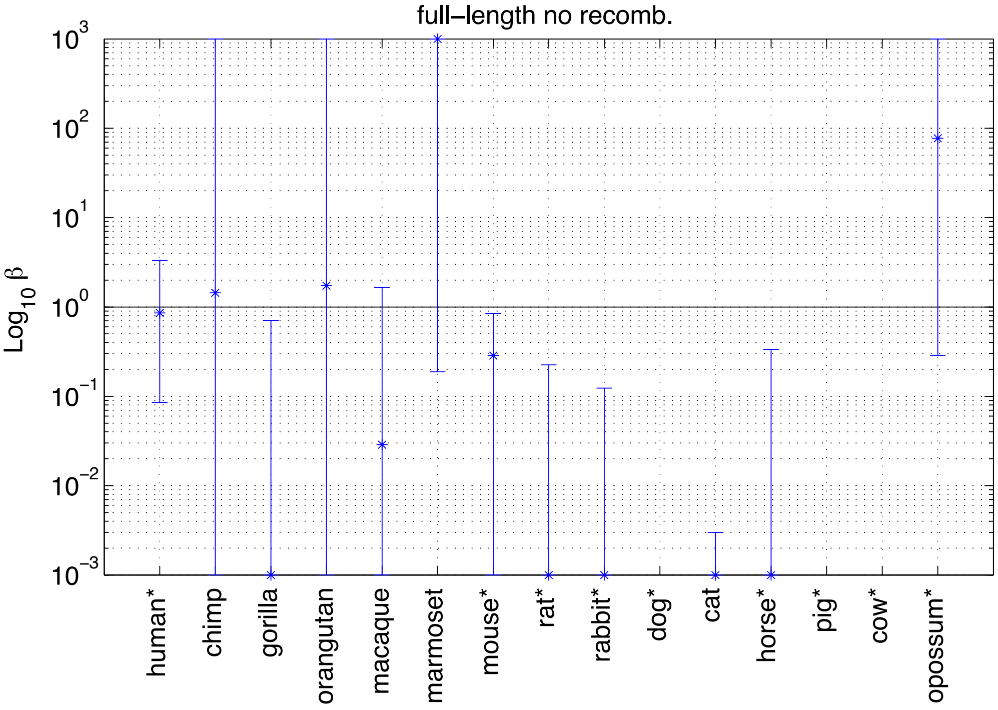

Figure 3: Point estimates and intervals on bias β implied by measurement of proviral distribution under the non recombination linked scenario. Points on the graph indicate the single bias β implied by our model. Error bars delineate the range of bias values under which we could expect to measure our observed ratios with a probability of less than 0.05. In those situations in which no point estimate or range of bias can be computed no markings appear. Asterisks beside species mark the genomes we consider as trustworthy and discuss in the Results section.
